# Influence of different application protocols of universal adhesive system on the clinical behavior of Class I and II restorations of composite resin – a randomized and double-blind controlled clinical trial

**DOI:** 10.1186/s12903-019-0913-3

**Published:** 2019-11-21

**Authors:** Andreia Assis Carvalho, Murillo Martins Leite, Jessica Karla Maia Zago, Carla Aparecida Bernardes Costa Meneses Nunes, Terezinha de Jesus Esteves Barata, Gersinei Carlos de Freitas, Érica Miranda de Torres, Lawrence Gonzaga Lopes

**Affiliations:** School of Dentistry, Federal University of Goiás, Praça Universitária, s/n, Faculdade de Odontologia, Setor Universitário, Goiânia-GO, CEP 74605220 Brazil

**Keywords:** Dental materials. Clinical trial. Dental bonding. Adhesives

## Abstract

**Background:**

Multimode adhesives incorporate the versatility of adapting to various clinical situations by its capacity to be used in different protocols. This study aimed to evaluate the clinical behavior of composite resin direct restorations (Class I and II) performed with different universal dentin adhesive application protocols comparing adapted FDI and adapted USPHS criteria.

**Methods:**

The current study is a randomized, double-blind, split-mouth, and convenience sample controlled clinical trial. The participants (age ≥ 18 years) had restorative need of Class I and/or II due to the presence of carious lesions and/or unsatisfactory restorations in at least three dental elements. Each participant received three application protocols for Scotchbond Universal adhesive (3M ESPE), one in each tooth to be restored: ER = etch-and-rinse + adhesive (n = 50); SEE = selective enamel etch + adhesive (n = 50) and SE = self-etch adhesive (n = 50). All teeth were restored in a similar way using Filtek™ Supreme composite resin (3M ESPE). Restorations were evaluated using the adapted FDI and adapted USPHS criteria, at baseline after 7 to 21 (12.02 ± 5.68) days (T1; n = 50 per group) and after 12 to 20 (15.8 ± 2.7) months (T2; n = 46 per group) by two previously calibrated evaluators (Kappa > 0.80). The statistical tests were performed between groups (Friedman), intragroup (Wilcoxon), and between the criteria considering acceptable and not acceptable restorations (McNemar), α = 0.05.

**Results:**

A statistically significant difference was observed only for the property “superficial staining”, between groups at T2 (*p* = 0.01) for ER (n = 13 with score 2 or more) and SEE (n = 3 with score 2 or more) and intragroup for ER (T1, n = 1 with score 2 or more; T2, n = 13 with score 2 or more, *p* = 0.001) and SE (T1, n = 0 with score 2 or more; T2, n = 8 with score 2 or more *p* = 0.007). For the other comparisons between groups, intragroup, and between the adapted FDI and adapted USPHS criteria, there were no statistically significant differences (*p* ≥ 0.05).

**Conclusions:**

It can be concluded that the different application protocols of the universal adhesive resulted in clinically “acceptable” restorations after 15.8 ± 2.7 months of follow-up. Adapted FDI and adapted USPHS criteria provided similar results to each other.

**Trial registration.**

Number in Brazilian Registry of Clinical Trials (ReBEC): RBR-9p3hdp. Registered 24 May 2015.

## Background

According to the form of demineralization of the dental substrate and the treatment given to the smear layer, the traditionally available adhesive systems can be divided into: etch-and-rinse (ER), self-etch (SE), and universal/multimode [1, 2]. ER systems profess the removal of the smear layer by etching of the dental substrate in a separate step from the application of the adhesive [1, 2]. SE adhesive systems are capable of modifying and incorporating the smear layer, simultaneously to dentin demineralization [3–5]. The universal adhesive can be used in different application modes: ER, SE, or selectice enamel etch (SEE) [6, 7]. The universal adhesive represents one type of one-step SE adhesives, and the durability and stability of bonded interfaces created by them continue to be questionable [8].

For a better comprehension of this process, it shall be understood that SE monomers are often less acidic than phosphoric acid, so that some minerals remain attached to the collagen fibers, allowing chemical bonds between the dental substrate and functional groups of the adhesive monomers [9]. Chemical bonding occurs with the interaction of functional acid monomers (carboxylic groups, phosphonic or phosphate) with hydroxyapatite crystals in dentin [10]. 10-methacryloyloxydecyl dihydrogen phosphate (10-MDP) is an example of a functional monomer that can interact with calcium ions in hydroxyapatite crystals in a process that is conducted until the formation of calcium salts of MDP (MDP-Ca salts) [10].

One of the advantages of using the universal adhesive in the clinical routine would be allowing the dental surgeon to choose the type of application protocol according to the clinical situation, optimizing the final result of the procedure [11]. However, while the development of universal adhesive systems has been an innovation in adhesive dentistry, it is questionable whether or not they are appropriate for all adhesive procedures [12]. Currently, there are only a few clinical trials that evaluate the bonding performance of universal adhesives [8]. There is still a lack of literature about these materials, with few clinical studies in Class V restorations [13–16].

It should be observed that the strength of the dentin bond is also variable according to the location of the cavity in the tooth (cervical or occlusal), and this is due to the variability of the dentinal tissue itself [17]. According to Purk et al. (2007) [18], the photopolymerizable composite resin may bond differently to the dentin, depending on the amount of cavity walls and there may be more cracks through the adhesive in the gingival wall than in the axial wall. Thus, clinical studies with universal adhesives performed in Class I and II cavities are necessary.

In this context, the clinical evaluation of restorations involves the use of criteria developed for some factors considered relevant in the clinical performance of dental restorative materials [19]. The evaluations can be judged according to different factors, among which are the USPHS (United States Public Health Service) criteria [19] and the FDI (*Fédération Dentaire Internationale*/World Dental Federation), being that the latter is divided into aesthetic, functional, and biological parameters of the restorations [20]. According to the FDI criteria, the restorations can be classified into five scores: 1 = clinically very good; 2 = clinically good; 3 = clinically sufficient/satisfactory; 4 = clinically unsatisfactory; and 5 = clinically bad [20]. According to the USPHS criteria, the restorations can be classified into three scores: Alpha, Bravo and Charlie [19]. Studies comparing the clinical behavior of different bonding strategies using modified FDI and USPHS criteria have concluded that FDI criteria are more sensitive than the modified USPHS criteria for small variations in clinical outcomes of noncarious cervical lesions [13, 14, 16].

Therefore, this work aimed to evaluate the clinical behavior of composite resin restorations performed with a universal adhesive system used in different application protocols comparing the adapted FDI and adapted USPHS criteria. Null hypotheses (is) were tested: (1) the adhesive application protocol (ER, SEE, or SE) does not influence the clinical behavior of Class I and II composite resin restorations over time, and (2) there is no difference between the adapted FDI and adapted USPHS evaluation criteria of the clinical behavior.

## Methods

### Study design

A randomized, double-blind and split-mouth clinical trial was conducted, which experimental design adhered to Consolidated Standards of Reporting Trials (CONSORT) guidelines. Clinical procedures were performed at the School of Dentistry of the Federal University of Goiás, Brazil, from June 2015 to April 2018. The research project was approved by the Research Ethics Committee (CAAE: 36829814.0.0000.5083), registered in the Brazilian Registry of Clinical Trials (ReBEC - http://www.ensaiosclinicos.gov.br; RBR-9p3hdp) and the participants signed a written informed consent form.

### Sample size

The sample size calculation was based on the retention rates, postoperative sensitivity, adaptation, and marginal coloration of the Scotchbond Universal adhesive (3M ESPE, St Paul, MN, USA) for Class V restorations after 18 months [14], using α of 0.05 and a power of 80%. Thereby, the number of 50 teeth per group was determined to detect a difference of 20% in the tested groups.

### Sample selection

Inclusion criteria were age equal to or older than 18 years; clinical and radiographic need for Class I and/or II restorations in posterior permanent teeth due to the presence of carious lesions and/or unsatisfactory preexisting restoration; presence of at least three teeth (one for each adhesive protocol) to be restored per participant, which antagonistic teeth were present; and at least 20 teeth in functional occlusion.

Exclusion criteria were periodontitis; users of braces or removable orthodontic appliances; removable prosthesis wearers; signs of parafunctional habits; pregnant or lactating women; and fluorosis in the enamel.

Radiographic examination included the use of interproximal and periapical digital radiographs as auxiliary means of diagnostic for participants who presented inclusion criteria during the clinical examination. Oral hygiene guidelines were given to all study participants prior to restorative procedures.

### Randomization and blinding

The randomization process was performed using manually generated tables by a member of the research group not involved in performing the clinical procedures. Details of the allocated group, including the adhesive protocol to be used and the teeth sequence to be restored, were written on sheets placed in sequentially numbered, opaque, sealed envelopes, which were opened at the time of the restorative procedure. The operator knew which type of intervention would be performed, however, the participants did not know which treatment they received for a given tooth, and the evaluators were also unaware of the adhesive protocols used (double-blind study).

The option for split-mouth study design [21] was performed so that the same participant could receive the three adhesive protocols to be evaluated.

### Restorative procedures

The clinical procedures were performed by two operators who are experts in restorative dentistry. One of them was responsible for implementing the operative procedures and the other was responsible for the restorative procedures. The basic composition of the main materials used in this study is described in Table 1.

**Table 1:** Composition of the main materials used in this study informed by the manufacturers

Material	Manufacturer	Lot	Composition*
*Condac 37* (Joinville, SC, Brazil)	FGM	090715	37% Phosphoric acid, thickeners.
*Filtek™ Supreme* (St Paul, MN, USA)	3M ESPE	N595157 (color B2E) 176149 (color B2B)	Bis-GMA, UDMA, TEGDMA, Bis-EMA, non-agglomerated silica, non-agglomerated and agglomerated zirconia, and aggregated particles of zirconia/silica.
*Scotchbond Universal Adhesive* (St Paul, MN, USA)	3M ESPE	565520	HEMA, MDP (10-methacryloyloxydecamethylene phosphoric acid), phosphate monomer, dimethacrylate, hydroxyethyl methacrylate, copolymer of polyalkanoic acid methacrylate, ethanol, water, initiators and silane.

*According to information from the manufacturers

With absolute isolation, the operative approach of carious lesion or unsatisfactory restoration was initially performed with spherical diamond tips (KG Sorensen, Cotia, SP, Brazil), of compatible number to carious lesion or unsatisfactory restoration, in high rotation under refrigeration (Kavo, Rio de Janeiro, RJ, Brazil), and then, the carious dentin were removed with carbide bur (Maillefer, Dentsply, Rio de Janeiro RJ, Brazil) in low-speed (Contra-angle, Kavo, Rio de Janeiro, RJ, Brazil). After cavity preparation, cavity cleaning was done with 2% chlorhexidine solution (Reymer from Brazil, Aparecida de Goiânia, GO, Brazil) with sterile cotton ball for 1 min.

Cavities considered very deep, the protection of the dentinopulpar complex was done with hydroxide of calcium cement (Hydro C, Dentsply, Rio de Janeiro RJ, Brazil) associated with resin-modified glass ionomer cement (Ionoseal, Voco, Cuxhaven, Germany), lining the bottom wall only. Deep cavities, the resin-modified glass ionomer cement was used. For medium or shallow depth cavities the adhesive protocols were applied directly. The Scotchbond Universal adhesive system (3M ESPE, St Paul, MN, USA) was applied to the tooth surface according to the drawn protocol, as detailed in Table 2.

**Table 2:** Clinical protocol of adhesive application in the three groups tested

Technique	Clinical sequence
Etch-and-rinse (ER)	- Total acid etching for 30s in enamel and 15s in dentin; - Abundant washing for 10s with water jet and removal of humidity excess with absorbent paper; - Application of the adhesive for 20s; - Air jet for 5s; - Photopolymerization for 20s.
Selective Enamel Etch (SEE)	- Selective acid etching in enamel for 30s; - Abundant washing for 10s with water jet and removal of humidity excess with absorbent paper; - Application of the adhesive for 20s; - Air jet for 5s; - Photopolymerization for 20s.
Self-Etch (SE)	- Application of the adhesive for 20s; - Air jet for 5s; - Photopolymerization for 20s.

The restorations were made with Filtek™ Supreme composite resin (3 M ESPE, St Paul, MN, USA) in the incremental oblique technique. Each increment was photocured for 20 s and a final polymerization of 40 s was done. Immediate finishing of the restorations was performed with fine diamond tips (KG Sorensen) and/or scalpel blades (Lamedid Comercial & Serviços Ltda, Barueri, SP, Brazil), after removal of absolute isolation and occlusal adjustment. The mediate finishing and polishing were done with silicone rubber tips (FlexiCups & FlexiPoints, Cosmedent, Chicago, IL, USA) in the medium (blue) and superfine (pink) granulations and felt disc (Diamond, FGM, Joinville, SC, Brazil) with diamond paste (Diamond Excel, FGM,, Joinville, SC, Brazil) at the first evaluation performed.

### Clinical evaluation

The first evaluation (T1) was performed 7 to 21 (12.02 ± 5.68) days after the restoration and, the second evaluation (T2) was performed after 12 to 20 months (15.8 ± 2.7) of monitoring. The evaluations were performed by two properly calibrated evaluators who were not involved with the restorative procedures. Excellent interexaminer agreement was obtained for all the criteria under analysis (Kappa> 0.80). Restorations were evaluated using the adapted USPHS (Table 3) [19] and adapted FDI (Table 4) criteria [20]. To evaluate the postoperative sensitivity of the teeth under analysis, air jets were applied for 10s with the triple syringe 2 cm from the occlusal surface [13, 14, 20], and the participant was asked if there was sensitivity caused by the air jet or if there was postoperative pain at some other time prior to the evaluation. Both examiners evaluated the restorations independently, and in the absence of agreement during the evaluations, consensus was sought before dispensing the participant.

**Table 3:** The adapted USPHS criteria with their categories and grading

Categories	Grading	“Decision”
Marginal discoloration	1. Alpha (clinically ideal) 2. Bravo (showing minor deviations from the ideal, nevertheless acceptable – except for retention and secondary caries) 3. Charlie (should be replaced to avoid future damage or requiring immediate replacement)	1. Acceptable (1, 2) 2. Not acceptable (3)
Fracture		
Retention		
Marginal integrity		
Postoperative sensitivity		
Recurrence of caries

**Table 4:** The adapted FDI criteria with their categories and grading

Categories	Sub-categories	Grading	“Decision”
A. Aesthetic properties	1. Surface lustre 2. Staining a. surface b. margin 3. Color match and translucency 4. Esthetic anatomical form	1. Clinically excellent/very good 2. Clinically good 3. Clinically sufficient/satisfactory (minor shortcomings, no unacceptable effects but not adjustable without damage to the tooth) 4. Clinically unsatisfactory/(but reparable) 5. Clinically poor (replacement necessary)	1. Acceptable (1, 2, 3) 2. Not acceptable (4, 5)
B. Functional properties	5. Fracture of material and retention 6. Marginal adaptation 7. Occlusal contour and wear a) qualitatively b) quantitatively 8. Approximal anatomical form a. contact point b. contour 9. Radiographic examination (when applicable) 10. Patient’s view		
C. Biological properties	11. Postoperative (hyper-)sensitivity and tooth vitality 12. Recurrence of caries (CAR), erosion, abfraction 13. Tooth integrity (enamel cracks, tooth fractures) 14. Periodontal response (always compared to a reference tooth) 15. Adjacent mucosa 16 Oral and general health		

The scores were dichotomized as “acceptable” for restorations that did not require intervention (clinically very good, clinically good, clinically sufficient/satisfactory, Alpha and Bravo) and “not acceptable” (clinically unsatisfactory, clinically bad, and Charlie) for those that needed repair or replacement [14]. To compare the adapted FDI and adapted USPHS criteria, considering the “acceptable” and “not acceptable” restorations, the properties of each criterion that could be compared were separated: adapted FDI (marginal staining, material fracture and retention, marginal adaptation, postoperative hypersensitivity and dental vitality, and recurrence of caries, erosion, or abfraction) and adapted USPHS (marginal discoloration, retention, fracture, marginal adaptation, postoperative sensitivity, and recurrence of caries).

### Data analysis

Statistical analysis followed the CONSORT protocol, considering the intention to treat, including all randomized participants and including those who did not participate in the evaluation appointments. Descriptive analysis was performed for the evaluated criteria. The Friedman test was used for the comparison between groups (ER, SEE, and SE) at T1 and T2. The Wilcoxon test was used for the intragroup comparison (i.e., each group between the times T1 and T2). The McNemar test was used for the comparison between the adapted FDI and adapted USPHS criteria considering acceptable and not acceptable restorations. All statistical tests were performed with SPSS software version 24 (IBM SPSS Statistics, Chicago IL, USA) at a significance level of 5% (α = 0.05).

## Results

**Fig. 1:**
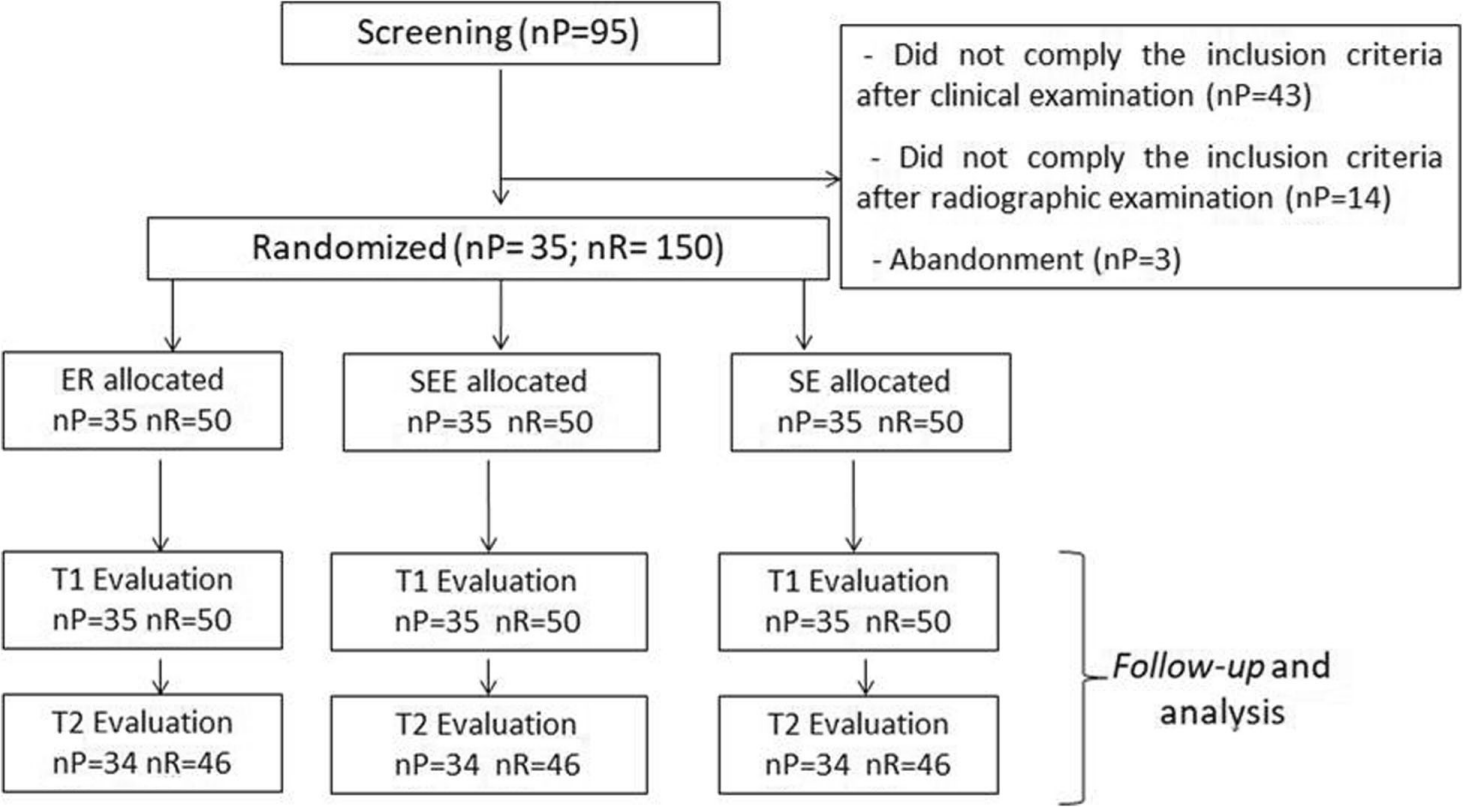
Characterization of the participants of the study (nP- number of Participants; nR- number of Restorations; ER- Etch-and-rise; SEE- Selective Enamel Etch; SE- Self-Etch; T1- first evaluation; T2- second evaluation)

Initially, 95 participants were evaluated. After screening, a final sample consisted of 35 subjects, accounting for total of 150 restorations (Fig. 1). Data corresponding to participants and to restored teeth are show in Table 5. The majority of participants were female (60%), 20–29 years old (77%), and the restored teeth were mainly molars (81%) with Class I type cavities (64%).

**Table 5:** Distribution of participants and restored teeth

Groups	Gender		Age (years)		Tooth		Arcade distribution		Type of preparation		Cavity Depth			
	M	F	20–29	30–50	PM	M	Jaw	Mand	I	II	S	M	D	VD
ER	14	21	27	8	10	40	33	17	34	16	15	4	16	15
SEE					8	42	29	21	30	20	19	1	18	12
SE					11	39	26	24	32	18	26	6	11	7
*A Freq*	14	21	27	8	29	121	88	62	96	54	60	11	45	34
*R Freq*	40%	60%	77%	23%	19%	81%	58%	42%	64%	36%	40%	7%	30%	23%

Abbreviations: *ER*- Etch-and-rinse; *SEE*- Selective Enamel Etch; *SE*- Self-Etch; *A Freq*-Absolute frequency; *R Freq-* relative frequency; *M*- male; *F*- female; *PM*- premolar; *M*- molar; Jaw- jaw; Mand- mandible; I- Class I; II- Class II; S- shallow; M- medium; D- deep; VD- very deep

Restorations were evaluated using the adapted FDI and adapted USPHS criteria 7 to 21 (12.02 ± 5.68) days after the restoration (T1; n = 50 per group) and after 12 to 20 (15.8 ± 2.7) months (T2; n = 46 per group) by two previously calibrated evaluators (Kappa > 0.80). The data obtained during the two-time evaluations (T1 and T2) are summarized in Table 6 (adapted USPHS criteria) and Table 7 (adapted FDI criteria).

**Table 6:** Number of restorations evaluated, for each experimental group, classified according to the adapted USPHS criteria at T1 and T2 times

	Scores	“Decision”	T1			T2		
			ER	SEE	SE	ER	SEE	SE
Marginal staining	Alpha	Acceptable	50	50	50	42	42	42
	Bravo		–	–	–	4	4	4
	Charlie	Not acceptable	–	–	–	–	–	–
Fracture	Alpha	Acceptable	50	49	50	46	46	45
	Bravo		–	–	–	–	–	1
	Charlie	Not acceptable	–	1	–	–	–	–
Retention	Alpha	Acceptable	50	50	50	46	46	46
	Bravo		–	–	–	–	–	–
	Charlie	Not acceptable	–	–	–	–	–	–
Marginal adaptation	Alpha	Acceptable	50	50	49	45	44	45
	Bravo		–	–	1	1	2	1
	Charlie	Not acceptable	–	–	–	–	–	–
Postoperative sensitivity	Alpha	Acceptable	47	47	47	46	46	46
	Bravo		–	–	–	–	–	–
	Charlie	Not acceptable	3^a,b,c^	3^d,e,f^	3^g,h,i^	–	–	–
Recurrence of caries	Alpha	Acceptable	50	50	50	46	46	46
	Bravo		–	–	–	–	–	–
	Charlie	Not acceptable	–	–	–	–	–	–

Abbreviations T1- first evaluation; T2- second evaluation; ER- Etch-and-rinse; SEE- Selective Enamel Etch; SE- Self-Etch. Letters overwritten: a.patient 29 tooth 16; b.patient 39 tooth 17; c.patient 46 tooth 36; d.patient 28 tooth 15; e.patient 39 tooth 36; f. patient 48 tooth 36; g. patient 8 tooth 16; h.patient 39 tooth 37; i.patient 49 tooth 46

**Table 7:** Number of restorations evaluated, for each experimental group, classified according to the adapted FDI criteria at T1 and T2 times

	D	*	T1			T2				D	*	T1			T2		
			ER	SEE	SE	ER	SEE	SE				ER	SEE	SE	ER	SEE	SE
Surface lustre	A	1	50	50	49	45	46	44	Approximal anatomical form contact point (nR Class II = 54)	A	1	14	19	16	11	14	12
		2	–	–	1	1	–	2			2	–	–	–	–	1	–
		3	–	–	–	–	–	–			3	2	1	2	3	2	4
	NA	4	–	–	–	–	–	–		NA	4	–	–	–	–	–	–
		5	–	–	–	–	–	–			5	–	–	–	–	–	–
Staining surface	A	1	49	50	50	33	43	38	Approximal anatomical form contour (nR Class II = 54)	A	1	15	20	16	13	16	14
		2	1	–	–	10	2	7			2	1	–	2	1	1	2
		3	–	–	–	2	1	1			3	–	–	–	–	–	–
	NA	4	–	–	–	1^a^	–	–		NA	4	–	–	–	–	–	–
		5	–	–	–	–	–	–			5	–	–	–	–	–	–
Staining margin	A	1	50	50	49	42	42	42	Patient’s view	A	1	50	50	49	46	46	46
		2	–	–	1	3	4	3			2	–	–	1	–	–	–
		3	–	–	–	1	–	1			3	–	–	–	–	–	–
	NA	4	–	–	–	–	–	–		NA	4	–	–	–	–	–	–
		5	–	–	–	–	–	–			5	–	–	–	–	–	–
Color match and translucency	A	1	47	46	46	42	41	41	Postoperative (hyper-)sensitivity and tooth vitality	A	1	47	47	47	45	46	46
		2	2	4	4	2	4	4			2	3	3	3	1	–	–
		3	1	–	–	1	1	1			3	–	–	–	–	–	–
	NA	4	–	–	–	1^a^	–	–		NA	4	–	–	–	–	–	–
		5	–	–	–	–	–	–			5	–	–	–	–	–	–
Esthetic anatomical form	A	1	49	47	47	45	42	43	Recurrence of caries (CAR), erosion, abfraction	A	1	50	50	50	46	46	46
		2	1	3	3	1	4	3			2	–	–	–	–	–	–
		3	–	–	–	–	–	–			3	–	–	–	–	–	–
	NA	4	–	–	–	–	–	–		NA	4	–	–	–	–	–	–
		5	–	–	–	–	–	–			5	–	–	–	–	–	–
Fracture of material and retention	A	1	50	49	50	46	46	45	Tooth integrity (enamel cracks, tooth fractures)	A	1	50	50	50	46	46	46
		2	–	1	–	–	–	1			2	–	–	–	–	–	–
		3	–	–	–	–	–	–			3	–	–	–	–	–	–
	NA	4	–	–	–	–	–	–		NA	4	–	–	–	–	–	–
		5	–	–	–	–	–	–			5	–	–	–	–	–	–
Marginal adaptation	A	1	50	50	49	45	44	45	Periodontal response (nR Class II = 54)	A	1	16	20	18	14	17	16
		2	–	–	1	1	1	1			2	–	–	–	–	–	–
		3	–	–	–	–	1	–			3	–	–	–	–	–	–
	NA	4	–	–	–	–	–	–		NA	4	–	–	–	–	–	–
		5	–	–	–	–	–	–			5	–	–	–	–	–	–
Occlusal contour and wear (qlt)	A	1	48	50	49	44	45	45	Adjacent mucosa	A	1	50	50	50	46	46	46
		2	2	–	1	1	1	1			2	–	–	–	–	–	–
		3	–	–	–	–	–	–			3	–	–	–	–	–	–
	NA	4	–	–	–	1^b^	–	–		NA	4	–	–	–	–	–	–
		5	–	–	–	–	–	–			5	–	–	–	–	–	–
Occlusal contour and wear (qtt)	A	1	49	50	50	45	46	46	Oral and general health	A	1	50	50	50	46	46	46
		2	1	–	–	1	–	–			2	–	–	–	–	–	–
		3	–	–	–	–	–	–			3	–	–	–	–	–	–
	NA	4	–	–	–	–	–	–		NA	4	–	–	–	–	–	–
		5	–	–	–	–	–	–			5	–	–	–	–	–	–

Abbreviations: *D*- Decision; *A*- Acceptable; *NA*- Not acceptable; *T1*- first evaluation; *T2*- second evaluation; *ER*- Etch-and-rinse; *SEE*- Selective Enamel Etch; *SE*- Self-Etch. *NA*- not applicable, *nR* - number of restorations. * Scores: 1- Clinically very good; 2- Clinically good; 3- Clinically sufficient/satisfactory; 4- Clinically unsatisfactory; 5- Clinically bad. Letters overwritten: a.patient 1 tooth 47; b.patient 21 tooth 26

For the “surface staining” property, a significant difference was observed at T2 between groups ER (n = 13 with score 2 or more) and SEE (n = 3 with score 2 or more; *p* = 0.01) and intragroup for ER (T1, n = 1 with score 2 or more; T2, n = 13 with score 2 or more; *p* = 0.001) and SE (T1, n = 0 with score 2 or more; T2, n = 8 with score 2 or more; *p* = 0.007). For the other comparisons between groups and intragroup, there were no significant differences (*p* ≥ 0.05).

In each group, postoperative sensitivity (initial assessment) was reported for three restored teeth, with “clinically good” (adapted FDI criteria) and “Charlie” (adapted USPHS criteria) scores. It is noteworthy that one participant reported postoperative sensitivity after the restorations with the three adhesive protocols tested. In the T2 assessment, no participant reported pain sensitivity in the period.

There was no significant difference when the adapted FDI and adapted USPHS criteria were compared (p ≥ 0.05), with “acceptable” restorations in adapted FDI (ER n = 44, SEE n = 46, and SE n = 46) and in adapted USPHS (ER n = 46, SEE n = 46, and SE n = 46) after 15.8 ± 2.7 months of follow-up.

## Discussion

The present study makes an important contribution to the literature on universal adhesive systems since it was performed in Class I and II cavities of permanent teeth, which differs from the studies of Mena-Serrano et al. (2013) [13], Perdigão et al. (2014) [14], Lawson et al. (2015) [15], and Lopes et al. (2016) [16] performed on Class V restorations. Considering that the most important parameter for evaluating the performance of an adhesive system is the retention [16], these studies justified the evaluation of Class V noncarious cervical lesions [13–16]. Nevertheless, the location factors of the cavities and type of masticatory forces could also influence the maintenance of the adhesive interface [17, 22], which highlights the importance of the evaluation performed here in Class I and II cavities.

Additionally, another factor that could interfere with the adhesive quality would be cavity cleaning after completion of the cavity preparation. In this study, 2% chlorhexidine solution was used before acid etching in the ER and SEE groups and prior to the application of the adhesive in the SE group, in order to disinfect the dentin surface, remove microorganisms, and minimize the possibility of recurrence of dental cavity [23]. However, chlorhexidine can also be used after etching, to minimize degradation by the metalloproteinases of collagen matrices that are incompletely infiltrated by the adhesive system [24]. There is no consensus in the literature concerning the efficacy of chlorhexidine use when applying SE adhesives or even when or how the chlorhexidine solution should be applied [25–27]. A recent clinical study with 36-month follow-up showed that cavity pre-treatment with chlorhexidine for inhibition of hybrid layer degradation does not add any beneficial effect to the clinical performance of restorations [28]. However, other laboratory studies have reported that for cavity cleaning prior to etching, chlorhexidine showed no significant change in microtensile bond strength of adhesive, even when the SE application mode was used [25, 29, 30]. Therefore, in the present work the option was to perform its prior application to the use of the adhesive system independently of its protocol.

In this context, although in the present study the application of the adhesive in a single layer was used, as indicated by the manufacturer, the possibility of its use in two layers is reported [31], in order to to enhance the bond strength. A laboratory work performed by Fujiwara et al. (2018) [31], the application of an universal adhesives in two layers improved the adhesive quality when compared to the application in a single layer. According to the authors of the study, this result may be related to the possibility of the double application protocol producing a more uniform adhesive layer and compensating for possible defects of single application [31]. This may be an important point of discussion for other long-term longitudinal follow-up clinical trials.

Based on the results obtained in this study, the first null hypothesis that “the adhesive application protocol (ER, SEE or SE) does not influence the clinical behavior of Class I and II composite restorations over time” had to be accepted, since the clinical behavior of composite resin restorations was similar, resulting in clinically acceptable restorations after 15.8 ± 2.7 months of follow-up, independently of the different application protocols of the universal adhesive (ER, SEE, and SE). Similar results to those reported by Mena-Serrano et al. (2013) [13] and Perdigão et al. (2014) [14] were obtained. These studies demonstrated that the clinical behavior of the Scotchbond Universal adhesive used in the form of SEE and SE, in Class V restorations, did not depend on the adhesive protocol tested in clinical follow-up of 6 and 18 months, respectively. These data may be related to the short period of clinical evaluation of these studies (6 and 18 months), which was similar to the present study (15.8 ± 2.7 months). In a study by Loguercio et al. (2015) [32], in 36 months of evaluation, there were signs of degradation when the universal adhesive was applied in the SE mode. Therefore, the restorations need to be re-evaluated after a long follow-up period (4–5 years) with the aim of confirming the results [33]. For Cuevas-Suarez et al., 2019 [8], the published data from the clinical trials suggested that the clinical performance of the universal adhesive did not depend on the bonding strategy in up to 36 months of follow-up.

Although statistically significant differences were observed for the surface staining property between T1 and T2 for the ER and SE groups and between ER and SEE techniques at T2, the scores remained within the clinically acceptable range. Possibly the observed surface staining was related to oral hygiene and eating habits of the participants, which was similarly observed by other authors [34, 35].

On the other hand, Lawson et al. (2015) [15], in an evaluation after 24 months, tested the Scotchbond Universal in the ER and SE technique and the Scotchbond Multi-Purpose (3 M ESPE) (ER) in Class V restoration and concluded that both tested adhesives deteriorated in relation to the adaptation and marginal discoloration. The authors also affirmed that Scotchbond Universal in the SE technique and Scotchbond Multi-Purpose presented similar clinical behavior, however, they stated that Scotchbond Universal with ER had the best results [15].

With the increase of the clinical evaluation period to 60 months, Baracco et al. (2016) [36] observed more frequent and severe marginal staining in Class I and Class II restorations when they tested the SE adhesive protocol (Adper Scotchbond SE and P90, 3 M ESPE) compared to the ER protocol (Adper Scotchbond XT, 3 M ESPE), as well as the presence of marginal adaptation deterioration in all groups according to the adhesive systems tested. Van Dijken and Pallesen (2017) [37], after 6 years of evaluation of Class II restorations, found failures related to fracture and recurrence of dental caries when comparing a three-step ER application mode adhesive system (cmf-els, Saremco AG) and an SE without HEMA (AdheSe One *F*, Ivoclar Vivadent). The failures corresponded to 11.4% of restorations with prior acid etching and to 20% in restorations with SE adhesive [37]. The authors inferred that the fracture of the restorations was related to the extension of the restorations [37]. In the present study, a case of fracture of the restoration was observed in the initial evaluation, which also suggests that the cause is due, probably, to the extension of the restoration. In in vitro study of Jacker-Guhr et al. (2019) [38], cohesive fractures were observed for ER, mainly in the enamel, probably due to higher bond strength using universal adhesives after additional phosphoric acid etching.

On the other hand, the present clinical trial differs from the studies of Mena-Serrano et al. (2003) [13], Perdigão et al. (2014) [14] and Loguercio et al. (2018) [33] in which the deterioration of the marginal adaptation of the restorations with the use of MDP universal adhesive was observed after 6 and 18 months of clinical evaluation, both for the adhesive protocol with ER and SE application modes. This difference may be related to the incidence of masticatory forces and complexity of the restorations, once the restorations were performed in Class I and II cavities, which is different from the cited works in which the restorations were performed in Class V cavities.

In a follow-up period of 18 months, Perdigão et al. (2014) [14] reported a 2.5% loss of retention in Class V restorations, and the universal adhesive protocol did not influence the loss of retention. In the 24-month follow-up of Class V restorations, retention rates of 87.6, 94.9, and 100% were reported for Scotchbond Multi-Purpose (ER), Scotchbond Universal in the SE application mode, and Scotchbond Universal with ER, respectively [15]. They concluded that Scotchbond Universal, in the SE and ER application mode, was similar to or better than Scotchbond Multi-Purpose [15]. In the present study, independently of the adhesive protocol applied, 100% retention of all the performed restorations was observed. This result should probably also be related to the location and type of the restorations (Class I and II). Although there was still no difference between the adhesive protocols, a systematic review showed that the enamel bond strength of universal adhesive is improved with prior phosphoric acid etching (SEE) [39]. SEE followed by the application of a mild universal adhesive currently appears to be the best choice to effectively achieve a durable bond to tooth tissues [8]. However, this effect was not evident for dentin with the use of mild adhesive with the ER [39].

In relation to postoperative sensitivity, in the present study there were no statistically significant differences between the groups, nor was there a relationship between postoperative sensitivity and cavity depth (47% shallow and medium/53% deep and very deep). The teeth that presented sensitivity at the baseline were classified with a “Charlie” score, since for the USPHS criterion there is no “Bravo” score for this criterion [19]. Thus, the restorations are classified as “Alpha” for no reports of postoperative sensitivity or “Charlie” for when there is a complaint. These patients were followed up, observing a progressive regression of sensitivity not requiring intervention. In the T2 evaluation, no provoked sensitivity was observed in all restored teeth. These results are in agreement with other works in the literature using the same adhesive system in the different etching protocols [13–15] and with other adhesive systems also in the mode with ER and SE [37, 40].

To compare the criteria, the properties evaluated were dichotomized as “acceptable” and “not acceptable”, similar to other studies [13, 14]. As there were no statistically significant differences for the comparisons between the adapted FDI and adapted USPHS criteria, the second null hypothesis that “there is no difference between the adapted FDI and adapted USPHS evaluation criteria of the clinical behavior” was also accepted. The USPHS and FDI clinical evaluation criteria are described in previous studies, such as the FDI being more sensitive than the USPHS in the “marginal adaptation” parameter [13, 14]. However, the option to maintain both criteria is to allow the comparison of results with studies that used only one of the criteria [16].

The clinical follow-up period of the present study may be considered a limitation, however, the divulgation of results is important for comparison with similar follow-up studies [14, 33]. Longitudinal clinical follow-ups for longer periods are necessary to evaluate the influence of different application protocols of the universal adhesive system (Scotchbond Universal) on the clinical behavior of Class I and II composite resin restorations. The datasets generated during the current study are available in the Federal University of Goiás Repository [41].

## Conclusions

It was concluded that the application protocols of the Scotchbond Universal adhesive system did not influence the clinical behavior of Class I and II composite resin restorations during the follow-up period (15.8 ± 2.7 months); and, the adapted FDI and adapted USPHS clinical evaluation criteria were similar to each other.

## Data Availability

The datasets generated during the current study are available in the Federal University of Goiás Repository [Federal University of Goiás Repository. Goiânia, Goiás. 2018. http://repositorio.bc.ufg.br/tede/handle/tede/8506. The dataset used is also available from the corresponding author on reasonable request.

## Abbreviations

10-MDP: 10-methacryloyloxydecyl dihydrogen phosphate
CONSORT: Consolidated Standards of Reporting Trials
ER: Etch-and-rinse
FDI: *Fédération Dentaire Internationale*/World Dental Federation
MDP-Ca salt: metacriloiloxidecil dihidrogênio fosfato-cálcio
ReBEC: Brazilian Registry of Clinical Trials
SE: Self-Etch
SEE: Selective Enamel Etch
T1: first evaluation
T2: second evaluation
USPHS: United States Public Health Service
